# Supplementary material for: Ribosome Pausing at Inefficient Codons at the End of the Replicase Coding Region Is Important for Hepatitis C Virus Genome Replication
Source: Int J Mol Sci. 2020 Sep 22;21(18):6955. doi: 10.3390/ijms21186955 (PMC7555993; doi:10.3390/ijms21186955)
Supplement: Supplementary file 1 [file ijms-21-06955-s001.pdf]

Supplement

Ribosome pausing at inefficient codons at the end of the replicase coding region is important for Hepatitis C Virus genome replication

Gesche K. Gerresheim[1§], Carolin S. Hess[1§], Lyudmila A. Shalamova[2], Markus Fricke[3], Manja Marz[4], Dmitri E. Andreev[5,6], Ivan N. Shatsky[5], and Michael Niepmann[1*]

1 Inst. of Biochemistry, Medical Faculty, Justus-Liebig-University, Giessen, Germany.
2 Inst. of Virology, Faculty of Veterinary Medicine, Justus-Liebig-University, 35392 Giessen, Germany.
3 Genevention GmbH, Göttingen, Germany.
4 RNA Bioinformatics and High Throughput Analysis, Faculty of Mathematics and Computer Science, Friedrich Schiller University Jena, Germany.
5 Lomonosov Moscow State University, Belozersky Inst. of Physico-Chemical Biology, Moscow 119234, Russia.
6 Shemyakin-Ovchinnikov Institute of Bioorganic Chemistry, Moscow 117997, Russia.
§ These authors contributed equally to this work.
* To whom correspondence should be addressed. Tel. +49-(0)641-9947471; E-mail: michael.niepmann@biochemie.med.uni-giessen.de. Address: Institute of Biochemistry, Faculty of Medicine, Justus-Liebig-University, Friedrichstrasse 24, 35392 Giessen, Germany.

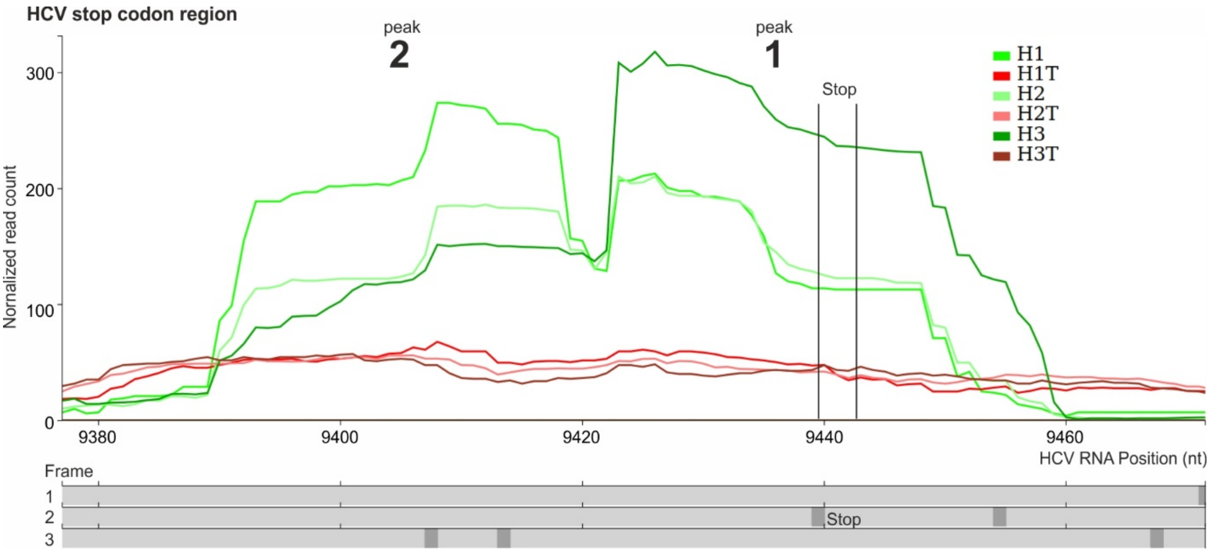

Supplementary Figure S1 (related to Fig 3A). Accumulated ribosome profiling reads in the ribosome peaks 1 and 2 at the HCV stop codon, separately displayed for the three replicates of the experiment (H1, H2, H3). Accumulated transcriptome reads (H1T, H2T, H3T) are shown as control. The position of the HCV stop codon is indicated.

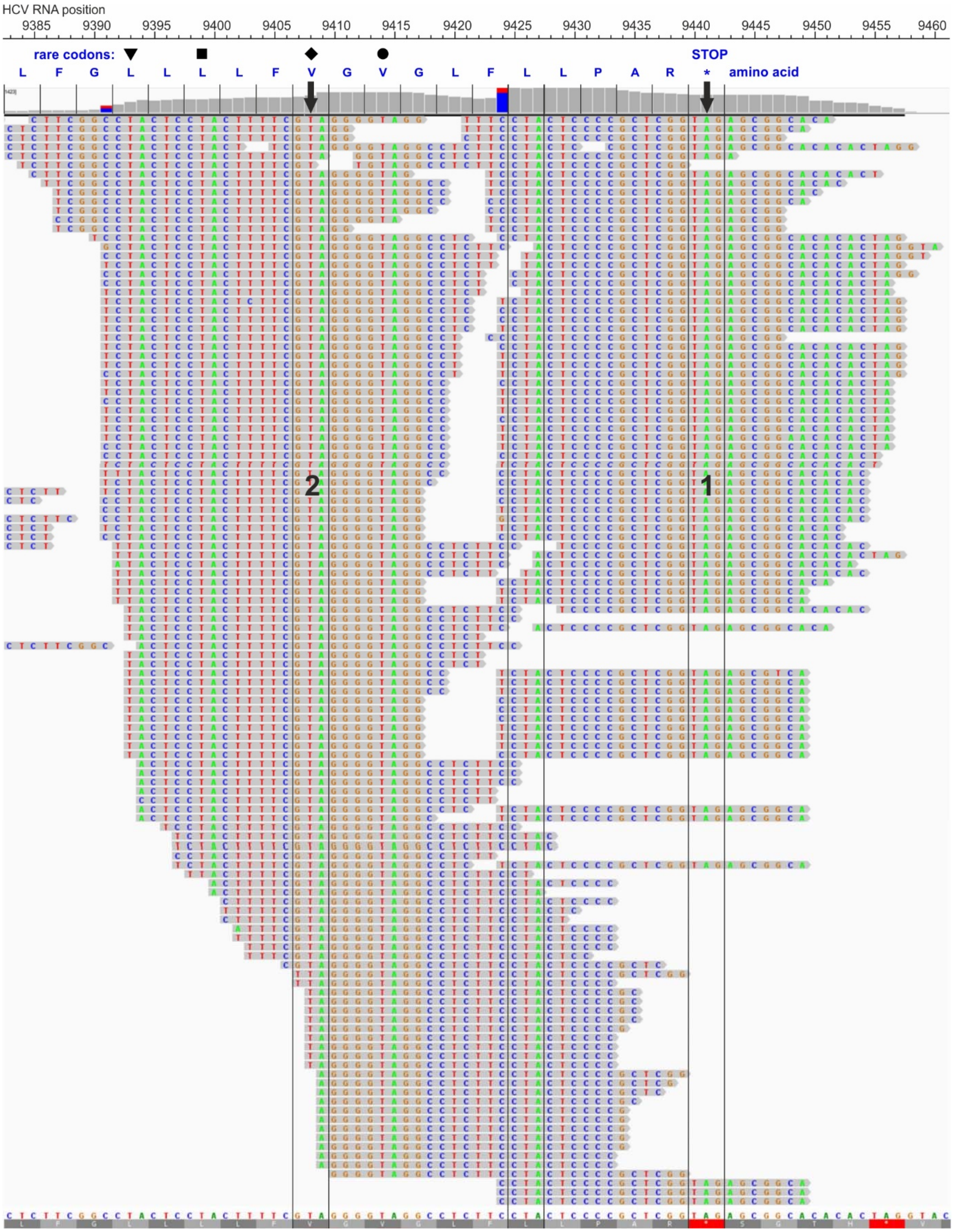

Supplementary Figure S2 (related to Fig 3B). Pausing of ribosomes upstream of the HCV RNA stop codon with sequence reads. The position of the stop codon and the most important upstream rare (low efficiency) codons are indicated by black arrows. The rare codons are indicated as in Figs. 2 and 4. The centers of the ribosomes placed in the peaks are indicated by thin vertical boxes.

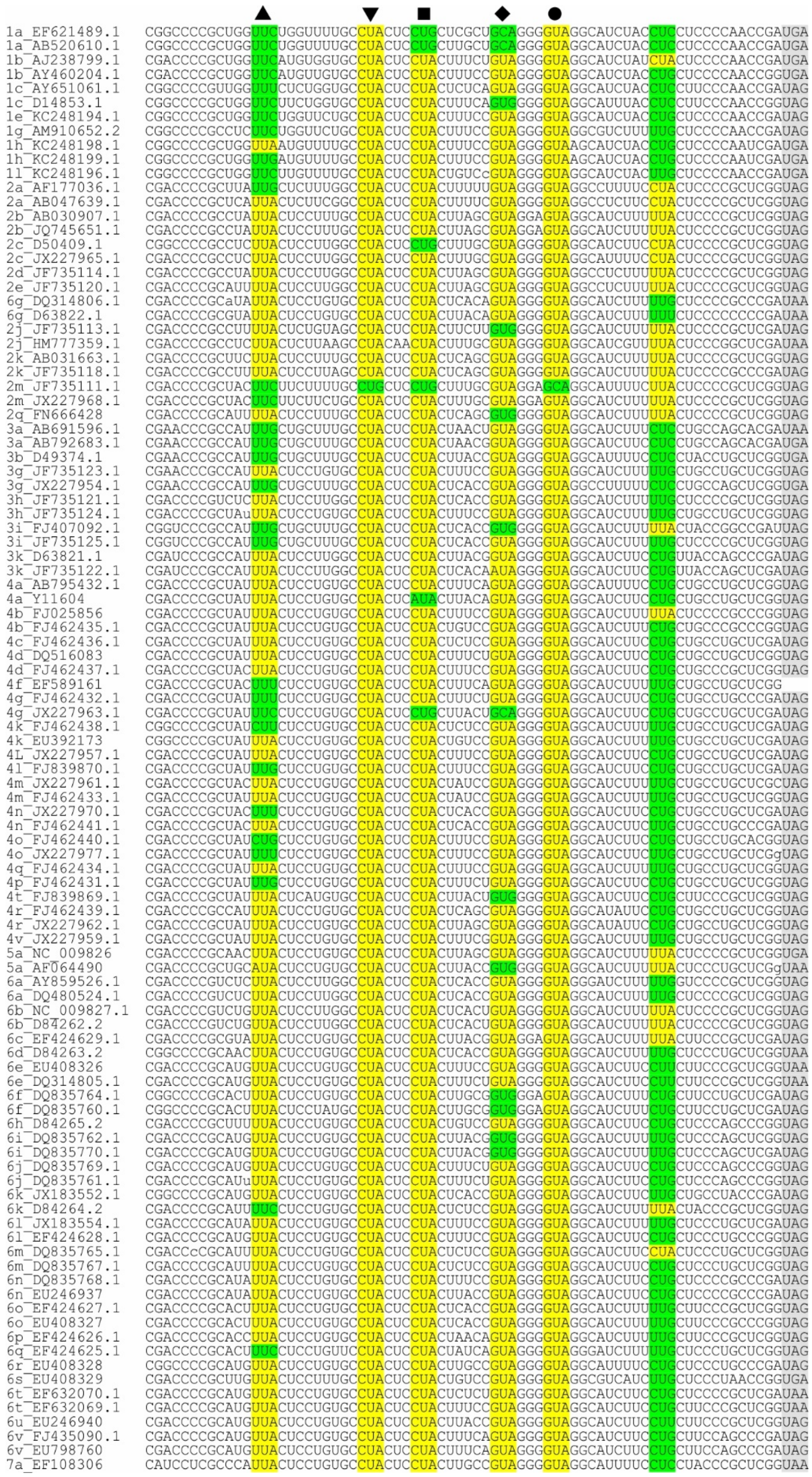

Supplementary Figure S3 (related to Fig 3D). Sequences at the 3′end of the NS5B coding region including the stop codon. Codons with moderate or high efficiency are marked in green, codons with low efficiency (rare codons) are shown in yellow.
